# Zingerone alleviates inflammatory pain by reducing the intrinsic excitability of anterior cingulate cortex neurons in a mice model

**DOI:** 10.3389/fphar.2025.1543594

**Published:** 2025-03-11

**Authors:** Yuanyuan Wang, Lang Dong, Shu Han, Yuehan You, Mingrui Zhang, Bingjing Sun, Hong Ni, Rongjing Ge, Jianhong Liu, Jiandong Yu

**Affiliations:** ^1^ Department of Neurosurgery, the First Affiliated Hospital of Bengbu Medical University, Bengbu, Anhui, China; ^2^ Laboratory of Brain and Psychiatric Disease, Bengbu Medical University, Bengbu, Anhui, China; ^3^ School of Basic Medicine, Bengbu Medical University, Bengbu, Anhui, China; ^4^ Department of Biochemistry and Molecular biology, School of Laboratory Medicine, Bengbu Medical University, Bengbu, Anhui, China; ^5^ Anhui Engineering Research Center for Neural Regeneration Technology and Medical New Materials, Bengbu Medical University, Bengbu, Anhui, China

**Keywords:** Zingiber officinale roscoe, analgesic, intrinsic excitability, complete Freund’s adjuvant (CFA), refractory period, electrophysiological, synaptic transmission

## Abstract

**Background:**

*Zingiber officinale Roscoe* has been shown to possess analgesic properties. Zingerone (ZO), a bioactive compound derived from Zingiber officinale Roscoe, exhibits a range of pharmacological effects, including anti-inflammatory, anti-cancer, antioxidant, antibacterial, and anti-apoptotic activities. However, the analgesic properties of zingerone remain unclear.

**Methods:**

Complete Freund’s adjuvant (CFA) was administered to the left hind paw of C57BL/6 mice to induce a model of inflammatory pain. The analgesic effects of zingerone were assessed using the Von Frey and Hargreaves tests. *In vivo* fiber photometry and whole-cell patch clamp techniques were employed to investigate the potential mechanisms.

**Results:**

Both acute and long-term treatment with zingerone resulted in a significant increase in mechanical and thermal pain thresholds in mice experiencing CFA-induced inflammatory pain. Mechanical stimulation led to a pronounced increase in calcium levels within the anterior cingulate cortex (ACC) neurons of the inflammatory pain model, which was alleviated by zingerone administration. Furthermore, zingerone was found to modify synaptic transmission to ACC neurons and decrease their intrinsic excitability by prolonging the refractory period of these neurons.

**Conclusion:**

Zingerone demonstrates potential for alleviating CFA-induced inflammatory pain by reducing the intrinsic excitability of ACC neurons in a mouse model.

## 1 Introduction

Pain is defined as an aversive sensory and emotional experience that is linked to actual or potential damage to bodily tissues. It is recognized as the fifth vital sign in human health and represents a significant challenge of global health (Raja et al., 2020). Inflammatory pain represents the most prevalent form of pain observed in clinical practice (Barr et al., 2015). Inflammatory pain significantly impacts patients’ quality of life and their capacity to work. Furthermore, the progression of acute inflammatory pain to chronic pain is likely to impose a substantial economic burden on both the patients’ families and society at large (Patnaik et al., 2017).

The ACC plays a crucial role in the integration of nociceptive perception and emotional responses associated with chronic pain (Baliki and Apkarian, 2015; Bliss et al., 2016; Rainville et al., 1997). In chronic pain, the ACC is identified as the most significantly activated region within the brain (Bushnell et al., 2013). The suppression of neuronal activity in the ACC has been shown to induce analgesic effects in the context of inflammatory pain (Pan et al., 2022). In chronic pain, there exist presynaptic and postsynaptic forms of long-term potentiation in the neurons of the ACC. A reduction in such potentiation is associated with the manifestation of analgesic effects (Koga et al., 2015; Zhuo, 2014). The enhancement of intrinsic excitability observed in chronic pain conditions is characterized by an increased input-output gain, as well as a reduction in spike threshold and refractory period (Yang et al., 2018). Both synaptic plasticity and intrinsic excitability plasticity play crucial roles in the hyperactivity of ACC neurons associated with chronic pain.

*Zingiber officinale Roscoe* has been shown to possess analgesic properties (Kim et al., 2022a). Among the 400 compounds identified in Zingiber officinale Roscoe, the primary bioactive components include gingerols, shogaols, and paradols (Mao et al., 2019). Recent studies have highlighted the analgesic effects of specific sub-components, particularly [6]-Shogaol and [6]-Gingerol (Kim et al., 2022a). These compounds exert their pain-relieving properties primarily through their anti-inflammatory actions in brain regions and the colon (Santos et al., 2024; Özdemir et al., 2023). For instance, [6]-Shogaol has been shown to significantly alleviate oxaliplatin-induced cold allodynia and mechanical hyperalgesia by modulating the serotonin system in the spinal cord (Kim et al., 2022b). However, current research has predominantly focused on the spinal cord, while chronic pain is often closely associated with emotional responses mediated by central brain regions. Whether other sub-components of *Z. officinale Roscoe* and central brain regions, particularly the ACC, play a role in its analgesic effects remains to be elucidated. Further investigation into the involvement of central brain mechanisms could provide a more comprehensive understanding of their therapeutic potential.

Zingerone exhibits a range of pharmacological properties, including anti-inflammatory, anti-cancer, antioxidant, antibacterial, and anti-apoptotic effects (Choi et al., 2018; Mahomoodally et al., 2021). Recent research has demonstrated that zingerone influences the electrophysiological properties of neurons. Specifically, zingerone has been shown to inhibit the amplitude of sodium and calcium currents, thereby modulating the functional activities of mouse pituitary cell lines (GH3 cells) and hippocampal neurons (Lai et al., 2022). Zingerone has the capacity to inhibit the pacemaker potential in a concentration-dependent manner through the modulation of ATP-sensitive potassium channels in gastrointestinal interstitial cells of Cajal (ICCs) (Kim et al., 2018). Zingerone has been shown to have the capacity to inhibit neuronal excitability. However, its potential analgesic effects through modulation of the ACC neuronal excitability remain to be elucidated. To investigate this, we employed a mouse model of inflammatory pain induced by complete Freund’s adjuvant, utilizing a combination of pain behavior assessments, calcium imaging, and patch-clamp techniques to evaluate the impact of zingerone on inflammatory pain and to explore the underlying mechanisms involved.

## 2 Method

### 2.1 Animals

110 male C57BL/6 mice, aged between 6 and 8 weeks, were procured from Henan Scobes Biotechnology Co., Ltd. (License No.: SCXK 2020-0005). The mice were maintained under a controlled environment with a 12-h light/dark cycle, temperature regulated between 20 and 26°C, and humidity levels maintained at 50% ± 20%. They were provided with *ad libitum* access to food and water and were acclimatized for a minimum of 1 week prior to the commencement of experimental procedures. All animal experimentation protocols adhered to the guidelines established by the Laboratory Animal Ethics Committee of Bengbu Medical University.

### 2.2 Inflammatory pain model

**FIGURE 1:**
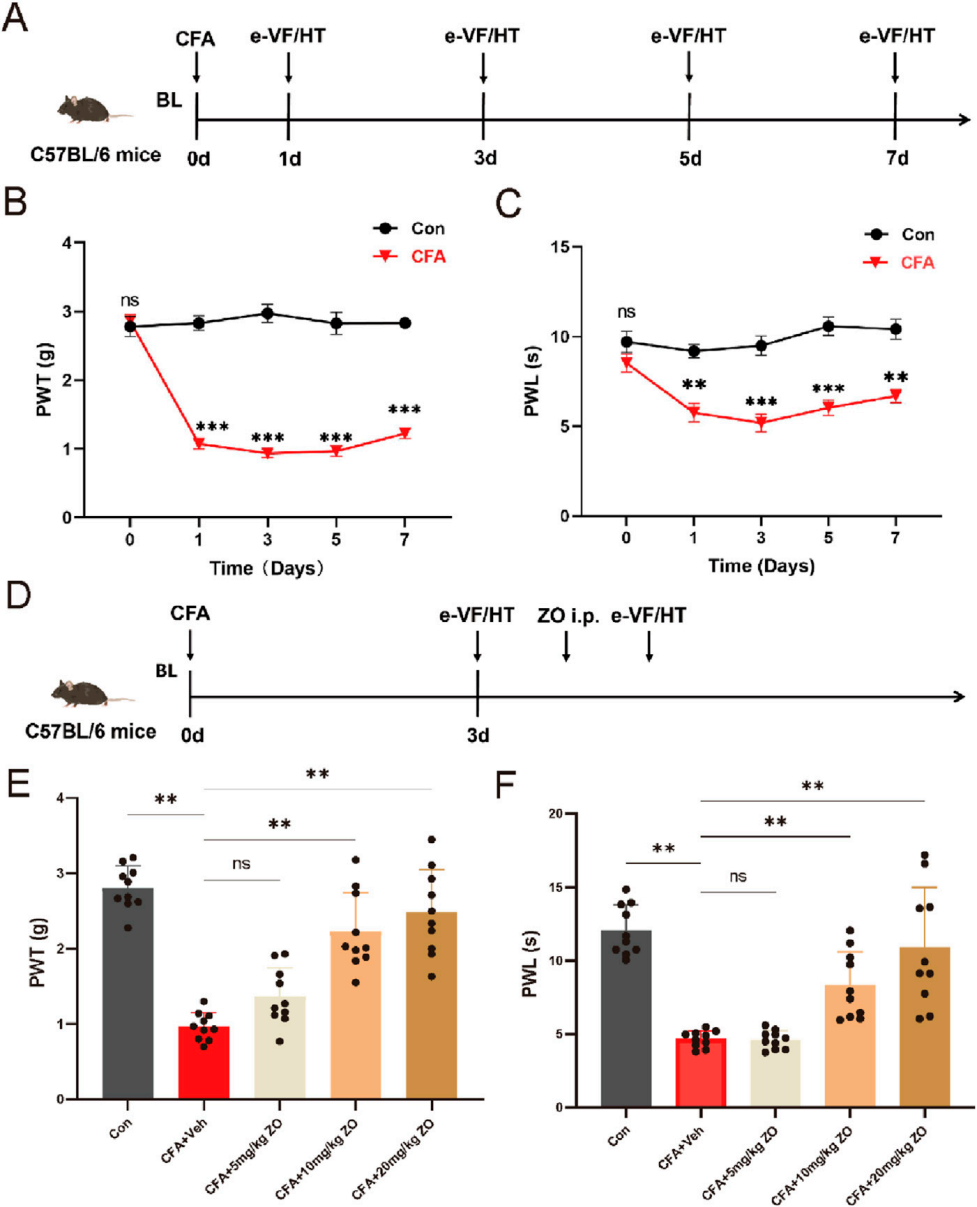
The acute administration of zingerone and its effects on hyperalgesia in a model of inflammatory pain induced by CFA. **(A)** The experimental timeline outlines the procedures employed in this study. **(B)** The paw withdrawal threshold (PWT) was assessed to evaluate mechanical allodynia following CFA injection (repeated measures two-way ANOVA with Bonferroni *post hoc* test, F (4,40) = 5.507, the p-value were calculated for the control and CFA groups on specified days). **(C)** The paw withdrawal latency (PWL) was measured to assess thermal hyperalgesia post-CFA administration (repeated measures two-way ANOVA with Bonferroni *post hoc* test, F (4,40) = 38.11, the p-values were calculated for the control and CFA groups on the specified days). **(D)** The timeline for evaluating the analgesic effects of zingerone is presented. **(E)** The PWT was utilized to assess the potential analgesic properties of zingerone in relation to mechanical allodynia (one-way ANOVA with Bonferroni *post hoc* test, F (4,45) = 35.16, P < 0.001). **(F)** The PWL was employed to evaluate the potential analgesic effects of zingerone on thermal hyperalgesia (one-way ANOVA with Bonferroni *post hoc* test, F (4,45) = 23.42, P < 0.001). All data are expressed as mean ± standard error of the mean (SEM). Statistical significance is indicated as *P < 0.05, **P < 0.01, and ***P < 0.001. The abbreviations used ns: not significant; i.p.: intraperitoneal injection; e-VF: electric Von Frey; HT: Hargreaves Test; Con: control; Veh: vehicle; ZO: zingerone.

**FIGURE 2:**
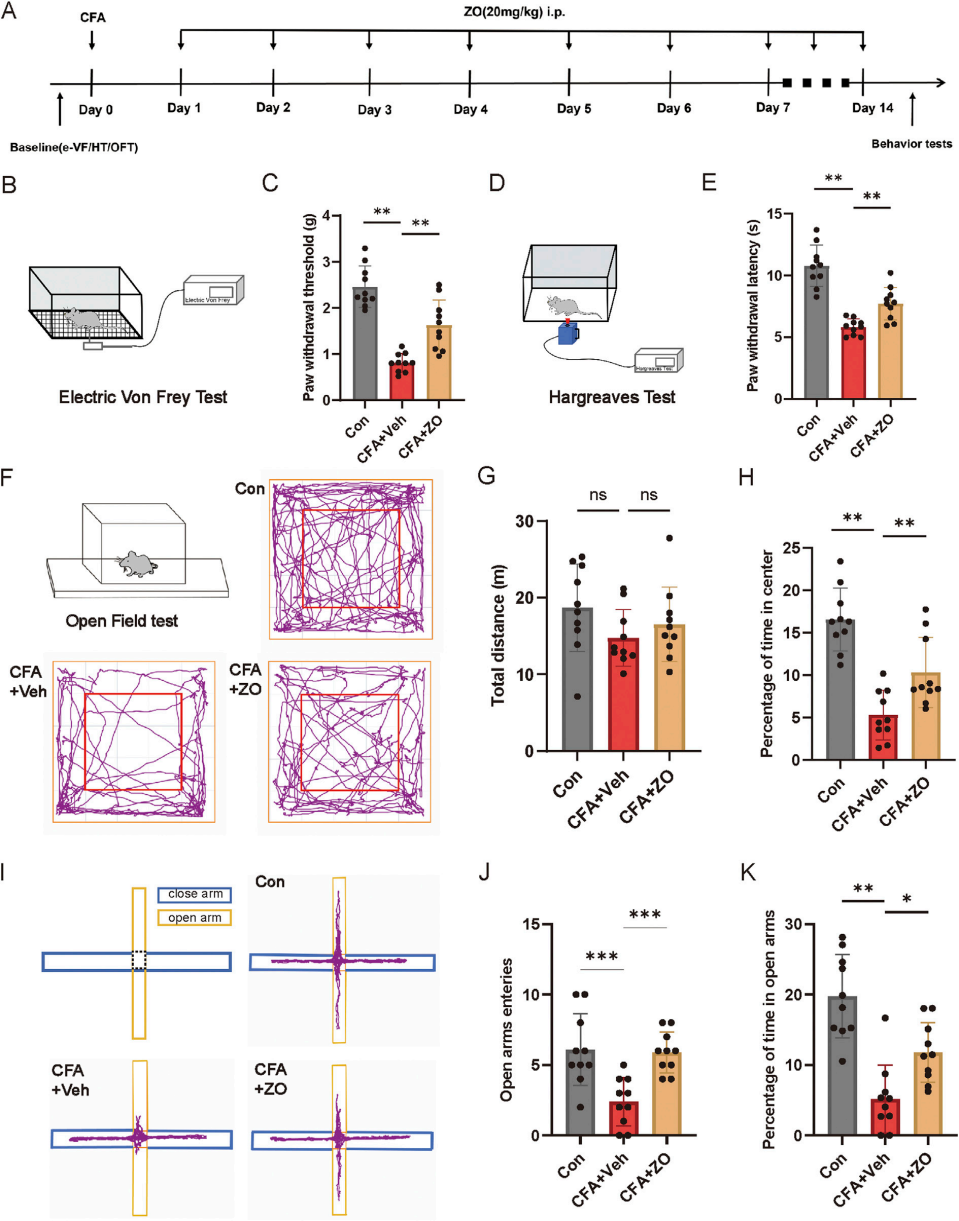
The prolonged administration of zingerone has been shown to mitigate chronic inflammatory pain and associated anxiety-like behaviors. **(A)** The experimental timeline is outlined. **(B, C)** The PWT was assessed to determine the long-term analgesic effects of zingerone on mechanical allodynia (one-way ANOVA with Bonferroni *post hoc* test, F (2,27) = 38.35, P < 0.001). **(D, E)** The PWL was evaluated to assess the long-term analgesic effects of zingerone on thermal hyperalgesia (one-way ANOVA with Bonferroni *post hoc* test, F (2,27) = 37.65, P < 0.001). **(F)** Locomotion patterns of mice subjected to control, CFA, and CFA combined with zingerone were recorded during an open field test. **(G, H)** Group data illustrating the total distance traveled (G, one-way ANOVA with Bonferroni *post hoc* test, F (2,27) = 1.696, P = 0.2) and the percentage of time spent in the center of the arena (H, one-way ANOVA with Bonferroni *post hoc* test, F (2,27) = 24.36, P < 0.001) during the open field test for the three experimental groups are presented. **(I)** Representative locomotion traces of mice injected with control, CFA, and CFA + zingerone in the elevated plus maze test are shown. **(J, K)** Group data reflecting the number of entries into the open arms (J, one-way ANOVA with Bonferroni *post hoc* test, F (2,27) = 11.22, P < 0.001) and the percentage of time spent in the open arms (K, one-way ANOVA with Bonferroni *post hoc* test, F (2,27) = 20.85, P < 0.001) during the elevated plus maze test for the three groups are provided. Data are expressed as mean ± SEM, with significance levels indicated as *P < 0.05, **P < 0.01, and ***P < 0.001. The abbreviations used ns: not significant; i.p.: intraperitoneal injection; e-VF: electric Von Frey; HT: Hargreaves Test; OFT: open field test; Con: control; Veh: vehicle; ZO: zingerone.

To induce inflammatory pain, 20 μL of CFA was administered subcutaneously to the plantar surface of the left hind paw of 66 mice (Alotaibi et al., 2023; Zhou et al., 2021). The control group (26 mice) received an equivalent volume of normal saline instead of CFA. After the induction of inflammatory pain, the affected mice were randomly divided into several treatment groups: 6 mice did not receive saline injection and served as the CFA group (Figure 1A); 10 mice received a saline injection 3 days after CFA administration and were designated as the CFA + vehicle group (Figure 1D); 10 mice received daily saline injections and were also labeled as the CFA + vehicle group (Figure 2A); and 40 mice were assigned to the CFA + ZO group. Within the CFA + ZO group, 10 mice were administered each of the differing doses of zingerone (Mani et al., 2017; Chopra et al., 2023; Mehrzadi et al., 2021) (as shown in Figure 1D), while another 10 mice received daily intraperitoneal injections of 20 mg/kg zingerone for 14 days (as shown in Figure 2A). The vehicle group received saline injections as a control. All behavioral assessments were conducted by an investigator blinded to the treatment allocations to ensure unbiased results.

### 2.3 von Frey test

The von Frey test was utilized to evaluate mechanical allodynia by determining the paw withdrawal threshold in response to mechanical stimuli, employing von Frey filaments (Zhongshi, Beijing, China). Prior to testing, mice were acclimatized in transparent plastic chambers equipped with a wire mesh floor for a minimum duration of 30 min. During the assessment, the measuring filament was positioned to penetrate the mesh and contact the plantar surface of the hind paw, with the force being gradually increased until a withdrawal response was elicited from the mice.

### 2.4 Hargreaves test

The Hargreaves test was employed to assess hyperalgesia by measuring the paw withdrawal latency, defined as the interval between the initiation of a light beam and the subsequent withdrawal of the paw. Mice were acclimatized in a manner identical to that used in the von Frey test. The thermal radiation source (Zhongshi, Beijing, China) was positioned beneath the center of the paw until a withdrawal response was observed from the animal.

### 2.5 Open-field test

Prior to the commencement of testing, the mice underwent a habituation period of 1 hour within the testing environment. Subsequently, each mouse was positioned in the center of a chamber measuring 50 × 50 × 30 cm. The mice were carefully placed in the chamber and observed via a camera for 5 minutes. The time spent by each mouse in the central area, defined as a 30 × 30 cm square, as well as the total distance traveled by the mice within the open field arena, were recorded. Additionally, a 75% ethanol solution was utilized to sanitize the open field arena between each trial.

### 2.6 Elevated plus maze test

The elevated plus maze apparatus was positioned 50 cm above the ground. It comprised two open arms measuring 25 × 8 × 0.5 cm and two closed arms measuring 25 × 8 × 12 cm, all extending from a central platform of dimensions 8 × 8 cm. The closed arms were enclosed by transparent walls that were 12 cm in height, while the open arms featured a 0.5 cm ledge. Each mouse was introduced into the center of the maze, oriented towards a closed arm. A camera mounted above the maze recorded the mouse’s movements during a 5-min observation period. The duration of time spent by the mice in both the open and closed arms was subsequently quantified. The maze was sanitized with 75% ethanol between each trial to ensure cleanliness. All behavioral assessments were conducted using ANY-maze (stoelting, United States) software for analysis.

### 2.7 Fiber photometry

The mice were subjected to anesthesia using pentobarbital administered intraperitoneally at a dosage of 75 mg/kg (Wang et al., 2023) and subsequently positioned on a stereotaxic apparatus (RWD, China). AAV vectors (rAAV-hSyn-GCaMp6s-WPRE, BrainVTA) were injected unilaterally into the ACC at the following coordinates: anteroposterior (AP) +0.98 mm, mediolateral (ML) ±0.30 mm, and dorsoventral (DV) −1.70 mm, at a rate of 20 nL/min. A ferrule fiber-optic cannula with a core diameter of 200 μm (RWD, China) was implanted above the site of viral injection and affixed to the skull using dental cement. Three weeks post-implantation, a multi-channel fiber photometry system (R811/821, RWD, China) was employed to monitor changes in calcium signaling during von Frey filaments (NC12775-99, North Coast, United States) stimuli. The change in fluorescence, denoted as ΔF/F, was calculated using the formula (Fsignal - F0)/F0, with F0 representing the baseline fluorescence, utilizing the data analysis module of the OFRS software (RWD, China).

### 2.8 Immunohistochemistry staining

Mice that received AVV injections were euthanized using pentobarbital administered intraperitoneally at a dosage of 75 mg/kg. Following euthanasia, the animals were subjected to a sequential perfusion process involving normal saline followed by 4% paraformaldehyde (PFA) in phosphate-buffered saline (PBS, at pH 7.2-7.4). The brains were subsequently harvested and post-fixed in 4% PFA overnight at a temperature of 4°C. After post-fixation, the brains underwent cryoprotection in a 10%–30% sucrose solution in PBS for a duration of 3 days. Staining of the 20 µm sections of mouse brain was conducted using DAPI (P24000042957, biosharp). Imaging was performed utilizing eclipse software (Nikon) on a D-Eclipse-C1 confocal microscope (Nikon).

### 2.9 Acute ACC slice preparation

Following administration of isoflurane anesthesia, the mice were promptly decapitated, and their brains were swiftly excised and immersed in ice-cold artificial cerebrospinal fluid (ACSF) (Zheng et al., 2022; Shao et al., 2023; Zhang et al., 2021), which comprised the following components: NaCl (124 mM), Glucose (12.5 mM), NaHCO_3_ (24 mM), KCl (2.5 mM), NaH_2_PO_4_ (1.2 mM), CaCl_2_ (2 mM), MgCl_2_ (2 mM), and HEPES (5 mM), adjusted to a pH of 7.35–7.45 and an osmolality of 310–330 mOsm/L. 300 μm coronal brain slices containing the ACC, were prepared using a vibrating microtome (Leica VT1000S). The slices were then rapidly transferred to oxygen-saturated ACSF at 33°C for a 30-min incubation period, after which they were maintained at room temperature until recording commenced.

### 2.10 Whole-cell patch-clamp recordings

Brain slices were recorded within a submersion chamber (Warner RC-26 G) that was perfused with oxygenated ACSF at ambient temperature (22°C–25°C). Whole-cell patch clamp recordings were performed on layer II/III pyramidal neurons in the ACC using infrared differential interference contrast (IR-DIC) optics (Nikon FN1). All whole-cell recordings were executed with a Multiclamp 700B amplifier (Molecular Devices, CA, United States) and sampled at a frequency of 10 kHz using a Digidata 1550 interface (Molecular Devices, CA, United States). The action potentials and spontaneous excitatory postsynaptic currents (sEPSCs) of ACC pyramidal neurons were recorded using a solution composed of: HEPES (10 mM), K-gluconate (140 mM), phosphocreatine (5 mM), EGTA (0.5 mM), Mg-ATP (4 mM), TrisGTP (1 mM), and NaCl (4 mM) at pH 7.40 and an osmolality of 290 mOsm. sEPSCs were recorded at a holding potential of −70 mV in the presence of SR 95531 (10 µM), a GABA_A_ receptor antagonist, within the ACSF. Additionally, spontaneous inhibitory postsynaptic currents (sIPSCs) of ACC pyramidal neurons were recorded using a solution containing: HEPES (10 mM), KCl (40 mM), K-gluconate (110 mM), phosphocreatine (5 mM), EGTA (0.5 mM), Mg-ATP (4 mM), TrisGTP (1 mM), and NaCl (4 mM) at pH 7.40 and an osmolality of 290 mOsm, also at −70 mV, in the presence of kynurenic acid (3 mM), an antagonist of ionotropic glutamate receptors, within the ACSF. Data analysis was conducted using Clampfit 10.7 software (Molecular Devices, CA, United States).

### 2.11 Statistical analysis

Statistical analyses and graphical representations were performed utilizing OriginPro 2019 (OriginLab Corporation, United States) and GraphPad Prism 8 (GraphPad Software, Inc., United States). Comparisons between two groups were executed using either paired or unpaired Student’s t-tests. For experiments involving multiple groups, after passed Shapiro-Wilk test, one-way or two-way analysis of variance (ANOVA) was employed, accompanied by Bonferroni or Tukey’s *post hoc* analyses. The data are presented as mean ± standard error of the mean (SEM), with differences deemed statistically significant at a threshold of *p < 0.05.

## 3 Result

### 3.1 Zingerone has been shown to mitigate inflammatory pain and associated anxiety-like behaviors

To establish a model of inflammatory pain in mice, CFA was administered via injection into the left hind paws (Figure 1A). Following the injection, mechanical allodynia (Figure 1B) and thermal hyperalgesia (Figure 1C) were observed, beginning 1 day post-injection and persisting for a minimum of 1 week. To assess the analgesic properties of zingerone on inflammatory pain, various doses of zingerone were administered intraperitoneally to the mice 3 days after the CFA injection (Figure 1D). A single administration of zingerone at doses of 10 mg/kg or 20 mg/kg led to a rapid reduction in both mechanical allodynia (Figure 1E) and thermal hyperalgesia (Figure 1F). To evaluate the long-term effects of zingerone (20 mg/kg), administration commenced 1 day post-CFA injection and continued for 14 days (Figure 2A). This treatment led to a significant decrease in both mechanical allodynia (Figure 2C) and thermal hyperalgesia (Figure 2E). Given that chronic pain can induce anxiety symptoms (Baliki and Apkarian, 2015), we further examined the impact of zingerone treatment on anxiety in CFA-injected mice. In the open field test, both control and CFA mice exhibited similar distances traveled (Figure 2G). However, CFA mice spent significantly less time in the center of the arena compared to control mice, a behavior that was reversed by zingerone treatment (Figure 2H). In the elevated plus maze test, CFA mice demonstrated reduced time spent in the open arms and infrequent entries into these areas compared to controls, with zingerone treatment also reversing this behavior (Figure 2J, K). Collectively, these findings indicate that zingerone effectively alleviates CFA-induced inflammatory pain and anxiety-like behaviors.

### 3.2 The neurons located in the ACC play a significant role in the analgesic effects of zingerone

**FIGURE 3:**
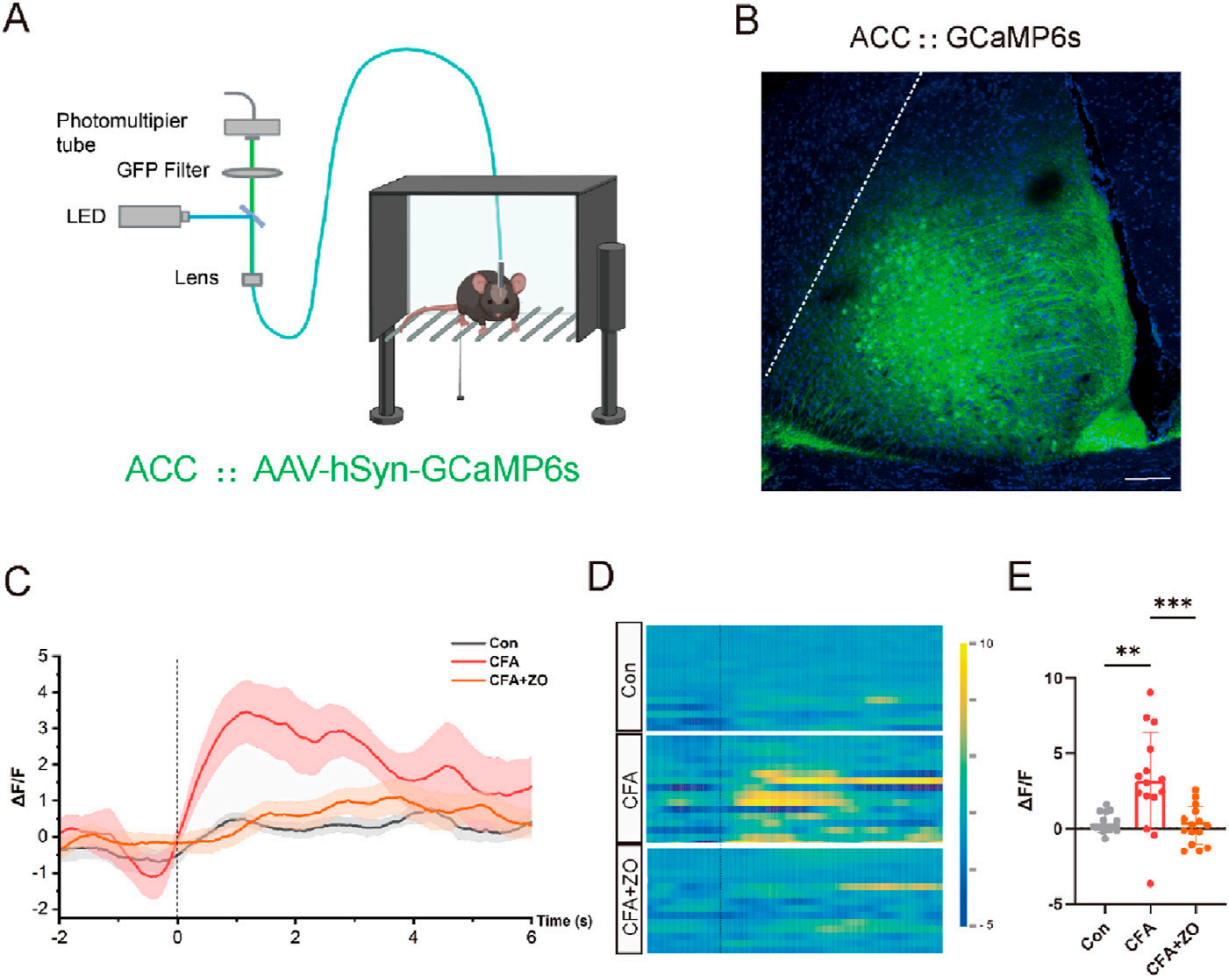
Zingerone reduces neuronal activity linked to pain in the ACC. **(A)** A schematic representation of the real-time fiber photometry assay is provided. Image was created with BioRender.com, with permission. **(B)** A representative image illustrating the expression of AAV-hSyn-GCaMP6s in the ACC is shown, with a scale bar of 100 μm. **(C, D)** The average calcium activity (C, “-2” refers to 2 s prior to stimulation) and corresponding heat maps **(D)** depicting calcium signals in response to 0.07g von Frey stimuli in control, CFA and CFA + zingerone mice are presented. **(E)** Statistics on the change in mean Ca^2+^ fluorescence signal in mice following stimulation (analyzed by one-way ANOVA with Tukey’s *post hoc* test, F(2, 42) = 9.961, **P < 0.01, ***P < 0.001). All data are expressed as mean ± SEM, with a sample size of n = 3 mice in each group.

The ACC plays a pivotal role in the perception of pain, with heightened neuronal activity in this region being closely associated with pain experiences (Pan et al., 2022). To assess the involvement of the ACC in the analgesic effects of zingerone, we conducted an investigation into the dynamics of neuronal activity within the ACC through fiber photometry recordings of calcium (Ca^2+^) signals (Figure 3A). An adeno-associated virus (AAV) encoding the fluorescent calcium indicator GCaMp6s (AAV-hSyn-GCaMP6s) was administered into the ACC of mice (Figure 3B). Three weeks post-injection, a notable increase in the fluorescence intensity of GCaMP6s-labeled neurons in the ACC was observed following stimulation of the ipsilateral hind limb using von Frey filaments in CFA mice. Following treatment with zingerone (20 mg/kg), this increase in fluorescence intensity was significantly diminished (Figures 3C–E). These findings indicate that zingerone treatment effectively mitigates alterations in neuronal activity within ACC neurons in a model of CFA-induced inflammatory pain.

### 3.3 Zingerone modifies synaptic transmission within the ACC

**FIGURE 4:**
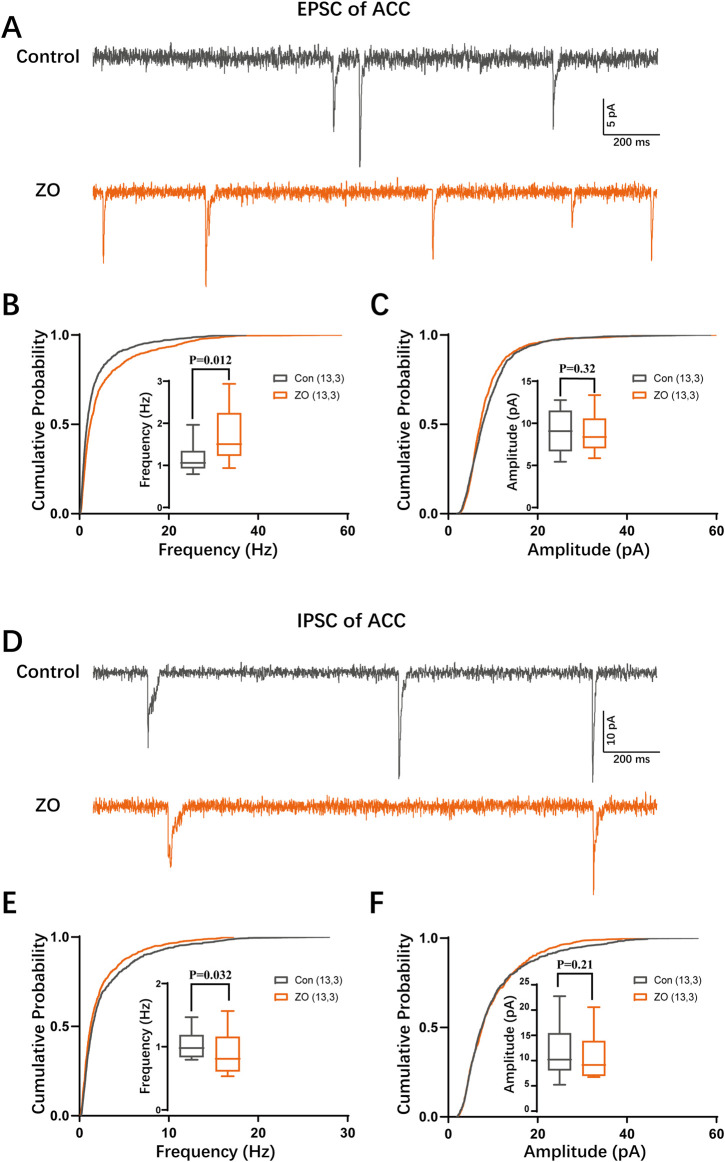
Zingerone modulates synaptic transmission in ACC neurons. **(A)** Representative traces of excitatory postsynaptic currents (EPSCs) recorded from ACC pyramidal neurons, both in the absence and presence of zingerone. **(B)** The cumulative frequency distribution and mean frequency of EPSCs in ACC pyramidal neurons are presented, comparing measurements taken prior to and following zingerone (10 μM) administration. **(C)** The cumulative amplitude distribution and mean amplitude of EPSCs in ACC pyramidal neurons are also analyzed before and after the application of zingerone. **(D)** Representative traces of inhibitory postsynaptic currents (IPSCs) are shown for ACC pyramidal neurons, recorded without and with zingerone. **(E)** The cumulative frequency distribution and mean frequency of IPSCs in ACC pyramidal neurons are evaluated before and after zingerone (10 μM) treatment. **(F)** The cumulative amplitude distribution and mean amplitude of IPSCs in ACC pyramidal neurons are assessed in relation to zingerone application.

To elucidate the mechanisms by which zingerone modulates the activity of the ACC neurons, we employed whole-cell patch clamp techniques to assess the synaptic inputs to these neuronal cells. Our findings indicate that zingerone (10 μM) administration (Lai et al., 2022) resulted in an increased frequency of excitatory postsynaptic currents (EPSCs) in ACC neurons (Figure 4A, B, control: 1.14 ± 0.09 Hz, ZO: 1.66 ± 0.16 Hz, 13 cells from three mice, paired T-test, P = 0.012), while the amplitude of EPSCs remained constant (Figure 4C, control: 9.21 ± 0.71 pA, ZO: 8.81 ± 0.65 pA, 13 cells from three mice, paired T-test, P = 0.32). Conversely, we observed a decrease in the frequency of inhibitory postsynaptic currents (IPSCs) following zingerone (10 μM) treatment (Figure 4D, E, control: 1.03 ± 0.06 Hz, ZO: 0.91 ± 0.09 Hz, 13 cells from three mice, paired T-test, P = 0.032), with the amplitude of IPSCs showing no significant change (Figure 4F, control: 11.86 ± 1.53 pA, ZO: 10.70 ± 1.19 pA, 13 cells from three mice, paired T-test, P = 0.21). These results suggest that zingerone has the capacity to modulate the frequency of both EPSCs and IPSCs in ACC neurons.

### 3.4 Zingerone has been shown to prolong the refractory period and reduce the intrinsic excitability of neurons within the ACC

**FIGURE 5:**
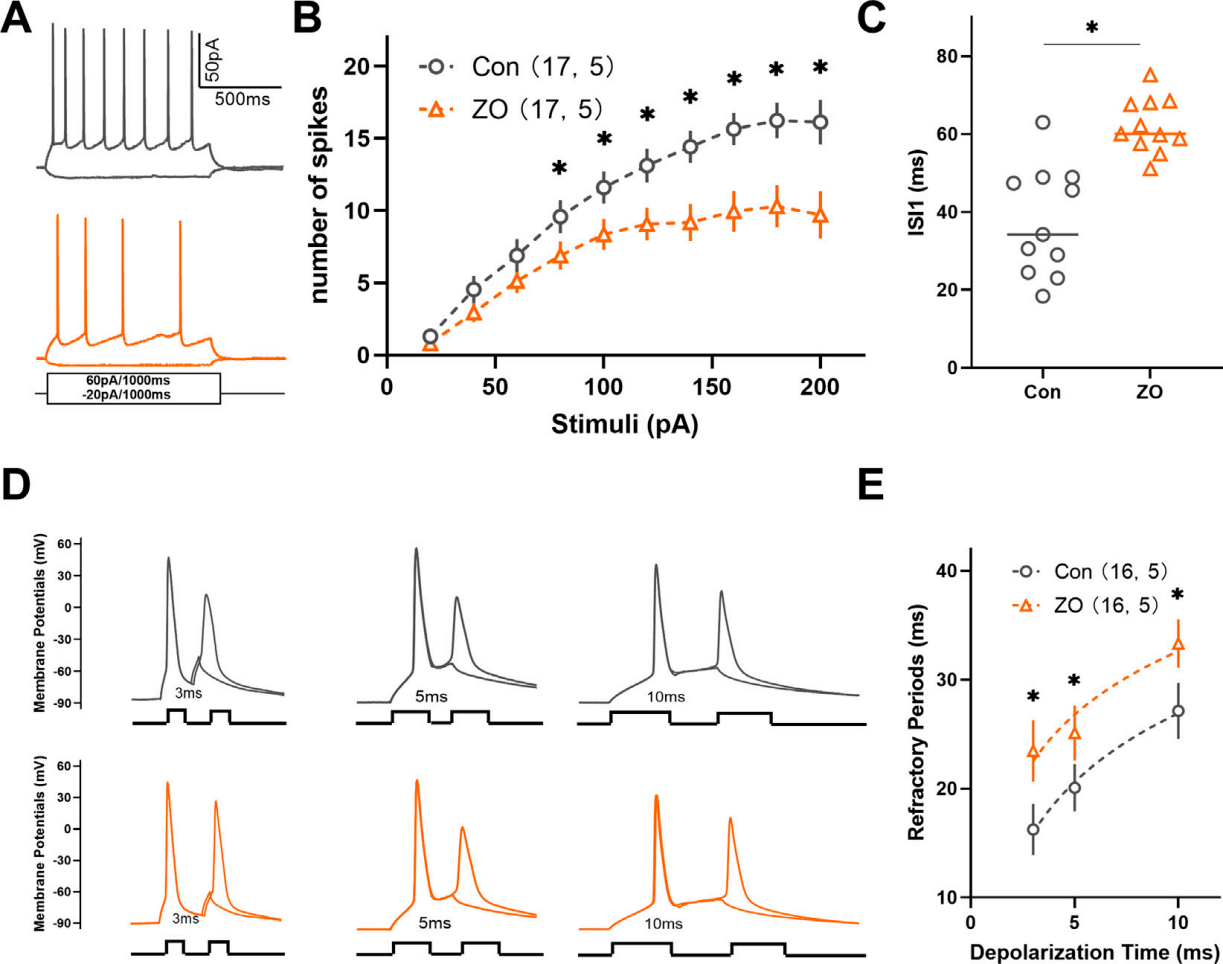
Zingerone has been shown to prolong the refractory period of action potentials and diminish the firing capacity of ACC neurons. **(A)** The representative firing patterns of neurons are depicted without zingerone (black, top) and with zingerone (orange, middle) in response to the stimulus waveform (black, bottom). **(B)** The application of zingerone (10 μM) resulted in a reduced gain in the input-output curve (repeated measures two-way ANOVA with Bonferroni *post hoc* test between groups, F (1, 320) = 53.3, P < 0.0001). **(C)** Furthermore, zingerone was found to increase the inter-spike interval (ISI) of ACC neurons (paired T-test, P < 0.05). **(D)** A comparison of the spike refractory periods of ACC neurons is illustrated, showing the absence of zingerone (black, top) versus its presence (orange, bottom). **(E)** Zingerone significantly decreased the spike refractory period in ACC pyramidal neurons (repeated measures two-way ANOVA with Bonferroni *post hoc* test between groups, F (1, 90) = 9.42, P = 0.003). Data are expressed as mean ± SEM, with significance levels indicated as *P < 0.05.

Neuronal intrinsic excitability plays a critical role in modulating neuronal activity. Consequently, we examined the impact of zingerone on the intrinsic excitability of ACC neurons. Our findings indicated a significant reduction in firing frequencies of ACC neurons in response to incrementally injected currents following zingerone (10 μM) perfusion (Figures 5A, B). Further analysis revealed that zingerone primarily prolonged the interval between the first and the second action potentials in response to 40 pA stimulation in ACC neurons (Figure 5C, control: 37.59 ± 4.21 ms, ZO: 62.18 ± 2.11 ms, 11 cells from five mice, paired T-test, P < 0.05). The refractory period, defined as the minimum interval between action potentials elicited by a specific testing stimulus, is a key determinant of neuronal firing rates. To further elucidate the effects of zingerone on the refractory period of ACC neurons, we varied the inter-pulse interval of depolarizing pulses. Our results demonstrated that zingerone (10 μM) significantly extended the refractory period of ACC neurons across various stimulus intensities (Figures 5D, E). These findings suggest that zingerone diminishes the intrinsic excitability of ACC neurons by prolonging their refractory period.

## 4 Discussion

**FIGURE 6:**
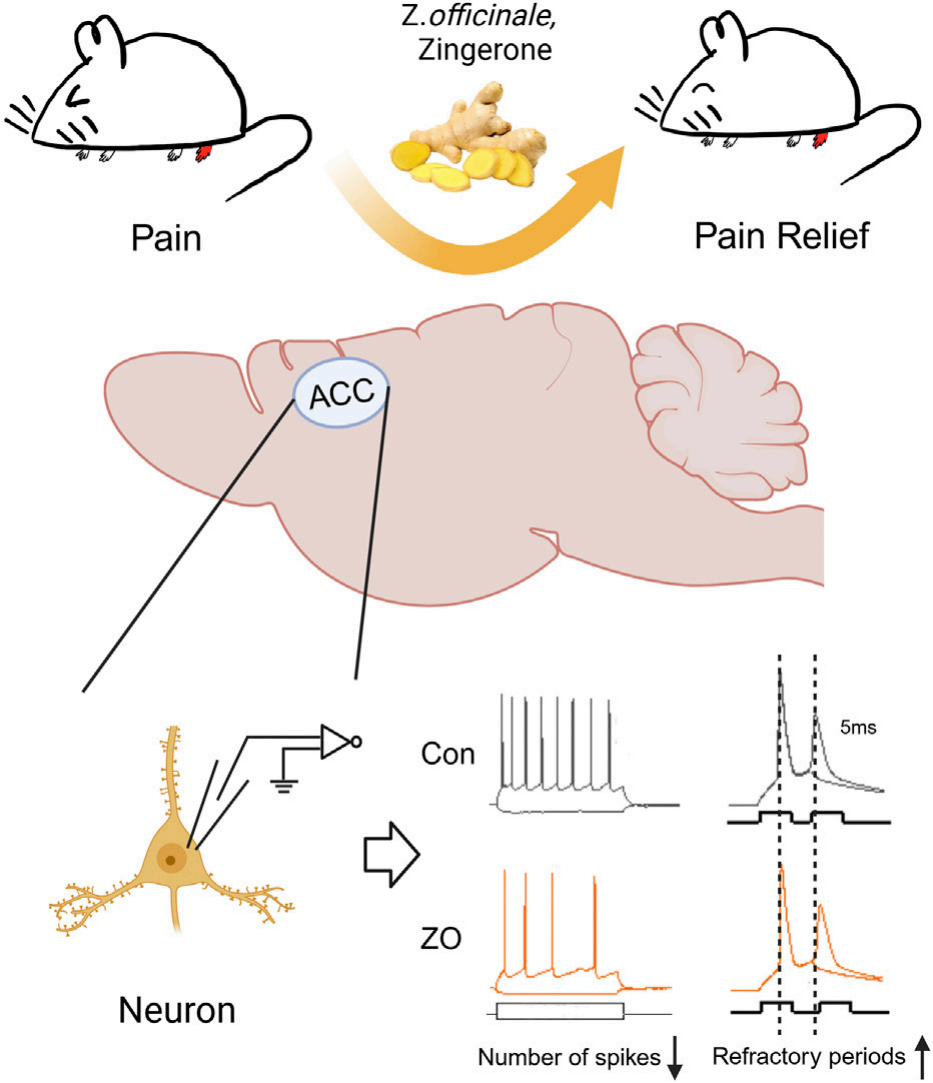
A schematic diagram of the zingerone alleviates inflammatory pain. Zingerone exhibits an analgesic effect in a mouse model of inflammatory pain by decreasing ACC neuronal intrinsic excitability through prolonging the refractory period of these neurons. Image was created with BioRender.com, with permission.

In the current investigation, we present novel findings indicating that zingerone exhibits analgesic effect in a model of inflammatory pain induced by CFA. This analgesic effect is attributed to zingerone’s modulation of synaptic transmission and the intrinsic excitability of neurons in the ACC (Figure 6).

Given the issues of tolerance, dependence, and withdrawal symptoms associated with commonly used clinical opioid analgesics like morphine, researchers have been actively seeking safer alternatives for pain management (Badshah et al., 2024; Lane et al., 2004; Little et al., 2013). Recent findings indicate that the naturally bioactive compound zingerone exhibits analgesic effect on inflammatory pain. Notably, zingerone has been documented to mitigate the development of antinociceptive tolerance and physical dependence associated with morphine, potentially through its capacity to reduce oxidative stress (Molavinia et al., 2024). Zingerone demonstrates anti-epileptic and neuroprotective properties through its various pharmacological activities, which encompass anti-inflammatory, antioxidant, and anti-apoptotic effects (Rashid et al., 2021). In our model, the analgesic effect is observed 30 min following the administration of zingerone, even in the absence of anti-apoptotic and anti-inflammatory mechanisms.

The ACC is a pivotal area involved in the perception of nociception, and the activity of pyramidal neurons within the ACC is elevated in both acute and chronic pain (Koga et al., 2010; Shyu et al., 2008). In this study, we utilized fiber photometry to monitor the activity of ACC neurons prior to and following the administration of zingerone during mechanical pain stimuli. This advanced technology allows for the real-time recording of calcium dynamics within neuronal populations in the ACC of awake mice while nociceptive behavioral tests are being conducted (Legaria et al., 2022; Gunaydin et al., 2014). Utilizing advanced technology, we have determined that zingerone diminishes the activity of ACC neurons in response to stimuli. Our findings align with those of a previous study, which demonstrated that targeted inhibition of pyramidal neuron activity in the ACC through optogenetic methods led to a rapid and significant reduction in hypersensitivity (Kang et al., 2015).

The excitability of a neuron is influenced by both synaptic inputs and its intrinsic properties. Prior research has demonstrated that inflammatory pain increases excitatory synaptic transmission while simultaneously decreasing inhibitory synaptic transmission in neurons of the ACC (Zhao et al., 2006; Gong et al., 2010). A separate investigation has demonstrated that the heightened intrinsic excitability of pyramidal neurons in the ACC is associated with neuropathic pain (Yang et al., 2018). In the current investigation, we performed a comprehensive analysis of the impact of zingerone on synaptic transmission and the intrinsic excitability of pyramidal neurons in the ACC utilizing the whole-cell patch-clamp methodology. Our research indicates that zingerone enhances the frequency of glutamatergic spontaneous excitatory transmission in pyramidal neurons of the ACC. Our findings are consistent with that zingerone has the potential to augment glutamatergic spontaneous excitatory transmission in neurons located in the spinal substantia gelatinosa of rats (Yue et al., 2013). Furthermore, zingerone appears to decrease the frequency of GABAergic spontaneous inhibitory transmission. Notably, our *in vivo* calcium dynamics recordings reveal that zingerone significantly inhibits neuronal activity in the ACC. Consequently, we hypothesize that the analgesic properties of zingerone may be attributed to its influence on intrinsic neuronal excitability rather than on synaptic transmission.

Following the administration of zingerone, there is an observed increase in the refractory period of pyramidal neurons in the ACC. This suggests that zingerone may play a role in modulating the activity of voltage-gated sodium channels (VGSCs). Notably, two constituents of ginger extract, namely [6]-gingerol and [6]-shogaol, have also been shown to inhibit VGSCs and exhibit analgesic properties (Hitomi et al., 2017). The VGSC has consistently served as a focal point for the development of analgesic pharmaceuticals, particularly those targeting subtypes expressed within the peripheral nervous system, including NaV1.7, NaV1.8, and NaV1.9 (Goodwin and McMahon, 2021). A new selective inhibitor of NaV1.8, designated as VX-548, is presently in phase III clinical trials aimed at further assessing its safety and efficacy for the management of post-surgical pain (Kaye et al., 2024). Zingerone has the potential to function as a natural inhibitor of VGSC for the purpose of analgesia. However, additional research is required to substantiate this claim.

Emerging evidence indicates temporal specificity in Notch signaling activation during neuropathic pain progression. In rodent models of neuropathic pain, the Notch pathway shows transient activation in the ACC during the early phase (7 days post-surgery) (Duan et al., 2021). Paradoxically, recent pharmacological studies demonstrate that both zingerone and its structural analog acetyl zingerone upregulate Notch pathway components (NOTCH1 and MAML3) in dermal fibroblasts (Swindell et al., 2020), suggesting potential tissue-specific regulation of this pathway. The molecular mechanisms underlying ACC neuronal sensitization appear predominantly mediated through the MAPK signaling cascade (Lin et al., 2021; Xiao et al., 2021). This critical pathway exhibits three principal therapeutic targets - p38, ERK, and JNK kinases - whose pharmacological inhibition in both neuronal and glial populations produces significant antinociceptive effects across various pain models (Deng et al., 2025; Li et al., 2023; Sanna et al., 2015). Of particular relevance, zingerone demonstrates multimodal regulation of MAPK signaling: mechanistic studies reveal its capacity to suppress NF-κB activation through concurrent inhibition of ERK, p38, and JNK phosphorylation in inflammatory models (Kim et al., 2010). Further research is needed to elucidate the precise molecular interactions and downstream effects associated with these pathways.

## 5 Limitations of the study

Despite the significance of our findings, this study has several limitations that should be acknowledged. First, the selection of the ACC as the focus was based on previous studies, but other brain regions, such as the ventrolateral periaqueductal gray (vPAG) (Liu et al., 2020; Baptista-de-Souza et al., 2020) and prelimbic cortex (PrL) (Gao et al., 2023; Wang et al., 2015), may also play a role in the antinociceptive effects of zingerone. Second, the conclusions drawn in this study are based solely on experiments conducted in male C57BL/6 mice. Given potential sex differences in pain processing and drug responses (Ram et al., 2021), the antinociceptive effects of zingerone in female mice, as well as in other animal models and humans, require further investigation. Third, the study lacks a detailed exploration of the molecular mechanisms underlying zingerone’s antinociceptive effects, as the analysis was limited to the cellular level. Elucidating the molecular mechanisms responsible for ACC neuronal hyperactivity could pave the way for the development of targeted therapies for chronic inflammatory pain.

## 6 Conclusion

The current investigation demonstrates that zingerone produces antinociceptive effects by inhibiting the activity of ACC neurons. The findings indicate that zingerone may represent a safe and promising therapeutic option for the treatment of migraines.

## Data Availability

The raw data supporting the conclusions of this article will be made available by the authors, without undue reservation.
